# Hypertensive Crisis in a Pediatric Patient Experiencing Clonidine Withdrawal

**DOI:** 10.1155/2022/9005063

**Published:** 2022-03-22

**Authors:** Can Cao, Matthew L. Lorenz, Phillip Sojka, Allison W. Brindle, Lisa Swartz Topor

**Affiliations:** ^1^The Warren Alpert Medical School of Brown University, Providence, RI, USA; ^2^Department of Internal Medicine, Rhode Island Hospital, Providence, RI, USA; ^3^Department of Pediatrics, Rhode Island Hospital, Providence, RI, USA

## Abstract

**Background:**

Clonidine, a central alpha-adrenoreceptor agonist, was initially developed as an antihypertensive. Though no longer commonly used for its original indication due to rebound hypertension after discontinuation, it is currently widely prescribed as a treatment for many pediatric indications including sleep disorders, behavioral concerns, and attention deficit hyperactivity disorder. *Case Report*. We describe a girl who developed prolonged symptoms of clonidine withdrawal, including hypertension and elevated serum metanephrines. *Discussion*. Clonidine withdrawal in pediatric patient can present with hypertensive urgency and other signs of sympathetic stimulation. Withdrawal can also lead to dramatic elevation in serum metanephrines. Treatment with a clonidine taper will reduce development of withdrawal symptoms.

**Conclusion:**

Given the rise in clonidine use in pediatric patients, clinicians should be aware of the risk of clonidine withdrawal and how to recognize and avoid its development.

## 1. Introduction

Clonidine, a central alpha-adrenoreceptor agonist, was approved in 1974 as a treatment for hypertension (HTN) in both adults and children [1]. Though clonidine is an effective antihypertensive agent [2–5], it is not favored clinically due to its adverse central nervous system (CNS) effects, multiple drug interactions, and potentially life-threatening withdrawal syndrome. [6–8]. Recently, clonidine has been increasingly used in pediatric patients for a variety of indications, including attention deficit hyperactivity disorder (ADHD), drug withdrawal, chronic pain, behavioral disorders, and sleep disturbance [9–11], with a significant rise in pediatric clonidine utilization between 2000 and 2014 [1, 9, 12]. Given the frequent use of clonidine and the array of clinical situations in which it may be used, recognizing the presentation and treatment of clonidine withdrawal is essential [1, 12].

Clonidine withdrawal can occur after its abrupt discontinuation. The symptoms of clonidine withdrawal arise from a compensatory dose-dependent increase in the release of norepinephrine following discontinuation of presynaptic alpha-2 receptor stimulation [13]. Additional theories include increased sensitivity to norepinephrine, decreased vagal tone, and increased activity of the renal-angiotensin system [14]. In rat models, chronic clonidine exposure causes downregulation of alpha-2 presynaptic autoreceptors and increased production of tyrosine hydroxylase, which both contribute to the long-lasting effects of clonidine withdrawal [13, 15]. Clonidine withdrawal classically presents with significant rebound HTN and signs of sympathetic overload, which can include tachycardia, restlessness, insomnia, headache, nausea, vomiting, palpitations, cardiac arrhythmia, and hypertensive encephalopathy [1, 16].

Here, we describe a patient who presented with a hypertensive crisis in the setting of multiple medical diagnoses and clonidine use for sleep disturbance.

## 2. Case Presentation

An 11-year-old female with autism spectrum disorder, epilepsy, behavior dysregulation, and sleep disorder presented to the emergency department (ED) with three days of urinary retention and vomiting in the context of increased constipation and poor oral intake. Home medications were clonidine 0.5 mg and melatonin 5 mg at bedtime and valproic acid twice daily. She missed at least two doses of each medication due to recurrent vomiting. She had also been prescribed polyethylene glycol and zonisamide, which her family had not been administering.

On arrival to the ED, her heart rate was 162 beats per minute and blood pressure was 164/118 mmHg (reference blood pressure, 95^th^ percentile for age and height: 120/77 mmHg) [17]. Her vital signs, exam, and initial workup were consistent with hypertensive urgency, moderate dehydration, and acute kidney injury, for which she received intravenous (IV) fluids and was admitted to the pediatric intensive care unit. Her blood pressure remained elevated despite treatment with hydralazine and isradipine, and later improved after restarting clonidine. Concerns about irregular home dosing of clonidine led to the decision to wean clonidine over the next 10 days. She was discharged home on amlodipine and a clonidine taper. The clonidine dose was 0.4 mg once daily at discharge, with instructions to reduce the clonidine dose by 50% every 3 days (clonidine 0.2 mg for 3 days, 0.1 mg for 3 days, 0.05 mg for 3 days, and then discontinued). Blood pressure was within target range prior to discharge home.

**Figure 1:**
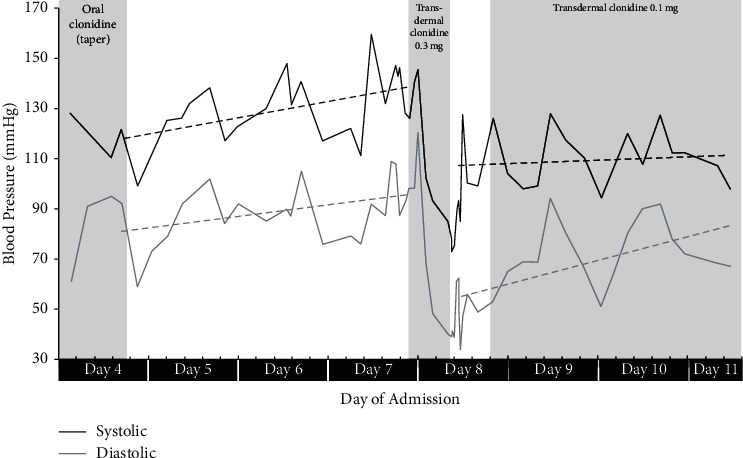
Blood pressure trends during second admission.

Two days after discharge, the patient developed intractable vomiting and inability to tolerate food or medications. She returned to the hospital with tachycardia and HTN and received IV fluids and hydralazine. HTN persisted following admission (Figure 1) despite treatment with hydralazine, isradipine, metoprolol, and labetalol; the clonidine taper continued per the prior plan. Her workup, which included unremarkable CT of the brain, abdominal ultrasound, and upper endoscopy, showed markedly elevated plasma metanephrine concentrations (Table 1), for which endocrinology was consulted.

The combination of her symptoms and elevated plasma metanephrines raised concern for clonidine withdrawal. Due to difficulty with oral intake, the oral clonidine was transitioned to transdermal formulation and a clonidine 0.3 mg transdermal patch was placed. This dose led to the development of hypotension, which improved following patch removal and treatment with IV fluids. Placement of a clonidine 0.1 mg transdermal patch led to resolution of vomiting, improved oral intake, and normalization of both blood pressure (Figure 1) and serum metanephrine concentrations (Table 1). She was discharged home with plans to taper the clonidine patch over several weeks; however, she later transitioned back to low-dose oral clonidine due to family preference.

## 3. Discussion

We describe severe and prolonged withdrawal symptoms in a child with autism receiving clonidine to treat disordered sleep and daytime behavioral problem. Though various reports exist for clonidine withdrawal in adults [2, 3, 7, 8, 18, 19], little has been reported on this syndrome in children. Given the rise in use of clonidine in pediatrics [1, 9–11], it is important to raise awareness of the dramatic presentation that can occur after abrupt cessation or rapid tapering of clonidine.

Although first line treatments of sleep disorders in children with autism center on behavioral techniques to promote sleep hygiene, medications are frequently trialed, especially when the quality of life of the patient and the family are significantly compromised. Melatonin and clonidine are often prescribed, although they do not always provide the desired effect [20–22]. In this case, the child had been prescribed both medications for several years and the family felt that they improved sleep and daytime behaviors.

Clonidine withdrawal in pediatrics presents similarly to what has been reported in adults. One study of children with Tourette syndrome described withdrawal from clonidine typically occurring within 72 hours of abrupt discontinuation and manifesting with symptoms lasting on average 3 days that included rebound HTN, tachycardia, and agitation [7, 23]. In contrast, the duration of severe clonidine withdrawal symptoms in our patient lasted longer, which may have been related to her high (0.5 mg) daily clonidine dose. A report of a child undergoing a 9-week wean from high-dose clonidine of 0.9 mg per day described asymptomatic tachycardia that lasted for 17 days [6]. Based upon these reports and our patient's symptoms, the starting dose and duration of the taper likely influenced the severity and duration of symptoms of clonidine withdrawal.

Our patient had elevated plasma metanephrine levels detected during her second hospitalization. Coupled with persistent HTN, the elevated metanephrine levels in our patient prompted consideration into whether pheochromocytoma or paraganglioma, rare catecholamine-secreting tumors that can cause extreme HTN [24, 25], was contributing to her symptoms. Other causes of elevated catecholamines that were considered included use of medications such as tricyclic antidepressants, antipsychotic agents, serotonin-reuptake and norepinephrine-reuptake inhibitors, and levodopa [26], use of stimulants such as caffeine and nicotine [27], and use of substances such as cocaine and its derivatives [28]. Mercury poisoning has also been reported to cause elevations in metanephrines [29]. Lastly, withdrawal from clonidine, ethanol, and benzodiazepines has also been associated with elevations in catecholamines [28].

Given that the hypertension and elevated metanephrines occurred while the clonidine taper was disrupted by vomiting and decreased oral intake, we considered that clonidine withdrawal was a likely etiology of the symptoms and laboratory findings, though further evaluation was warranted. She was not treated with any other medications associated with elevated catecholamines, did not drink alcohol or use illicit substances, and had no history of mercury exposure.

As clonidine does not affect autonomous tumor activity, clonidine administration can be used as a pharmacologic test to evaluate for pheochromocytoma [30]. Our patient's plasma metanephrines normalized with clonidine treatment and remained in the target range on repeat measurement, effectively ruling out pheochromocytoma (Table 1), as the elevated metanephrines in pheochromocytoma would not normalize after treatment with clonidine. In clonidine withdrawal, it is hypothesized that the release of drug-related catecholamine suppression causes a surge in circulating levels of catecholamines, contributing to the classic withdrawal symptoms and elevated serum metanephrine levels [4, 8, 23]. Evidence of this surge in actively withdrawing patients has been reported previously [8, 23].

Treatment of acute clonidine withdrawal involves controlling the symptoms of hypertensive crises as well as restarting clonidine with a slow taper [3, 18]. While there are no formal guidelines for tapering clonidine in children, previous literature suggests that slower and longer tapers, especially for patients on high maintenance doses, are better for symptomatic control of clonidine withdrawal [4, 6, 16]. Earlier administration of clonidine improves HTN in patients experiencing acute withdrawal symptoms [16]. Avoidance, or early recognition, of clonidine withdrawal can streamline clinical management, making the syndrome an important consideration in cases of acute pediatric HTN.

## Figures and Tables

**Table 1:** Selected laboratory results during second admission.

Laboratory Test	Reference range	Day 1	Day 7	Day 11
Total catecholamines	≤205 pg/mL	726	128	118
Metanephrine	≤57 pg/mL	107	31	51
Normetanephrine	≤148 pg/mL	619	97	67

## Data Availability

The data used to support the findings of this study are available from the corresponding author upon request.

## Abbreviation

CNS: Central nervous system
ED: Emergency department
HTN: Hypertension
IV: Intravenous.
